# CASE REPORT Laser-Assisted Indocyanine Green Evaluation of Paramedian Forehead Flap Perfusion Prior to Pedicle Division

**Published:** 2013-02-18

**Authors:** Ajul Shah, Alexander Au

**Affiliations:** Section of Plastic and Reconstructive Surgery, Yale University School of Medicine, New Haven, Conn

## Abstract

**Objective:** To present the use of indocyanine green and the LifeCell SpyElite System to confirm perfusion and viability of a forehead flap prior to division and inset, thereby eliminating the question of flap survival based on clinical judgment alone. **Methods:** A case report of a 67-year-old man with a forehead flap reconstruction following an acquired nasal defect due to resection of an adenoid cystic carcinoma is presented. LifeCell SpyElite System was used to confirm perfusion prior to pedicle division. **Results:** The LifeCell SpyElite System was used to confirm perfusion to the forehead flap prior to pedicle division during the second stage of the procedure. After confirming perfusion, the pedicle was divided and the flap was inset. The remainder of the patient's operative and postoperative course was uneventful, and he healed without incident. **Conclusions:** Indocyanine green and the LifeCell SpyElite System is a reliable method to confirm perfusion and viability of staged reconstructive procedures prior to division and inset, thereby eliminating the question of flap survival based on clinical judgment alone. This is a practical application of this technology that has not been previously described in the plastic surgery literature.

Indocyanine green (ICG) angiography allows real time evaluation of flap perfusion intraoperatively and is rapidly being applied to a number of different reconstructive procedures.^1^^-^4 We present the use of ICG and the LifeCell SpyElite System to assess forehead flap perfusion and viability prior to pedicle division during staged nasal reconstruction. This is a practical application of this technology that has not been previously described in the plastic surgery literature.

## CASE REPORT

A 67-year-old gentleman with a 30-year history of chewing tobacco underwent excision of nasal polyps in December 2011. Final surgical pathology revealed adenoid cystic carcinoma, and he subsequently underwent bilateral lateral rhinotomies, resection of skull base tumor including nasal bones, frontal process of the maxilla, and nasal septum. After intraoperative evaluation of the defect, the defect was reconstructed with a pedicled mucosal lining flap, split calvarial bone graft, and paramedian forehead flap.

**Figure 1:**
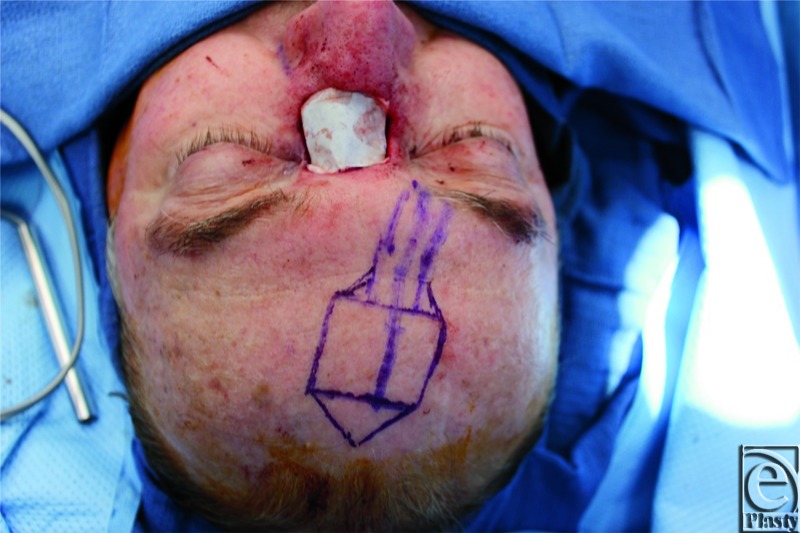
Template of defect and proposed reconstruction.

**Figure 2:**
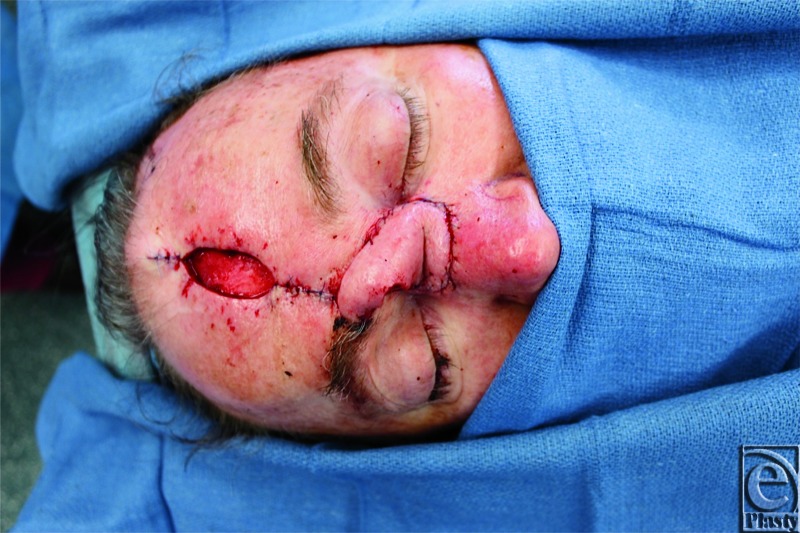
Transposed forehead flap.

The design of the paramedian forehead flap was based on a paper template that was created from the defect. A Doppler probe was used to identify the course of the right supratrochlear vessels (Fig 1). The flap was raised from distal to proximal, first in the supra-periosteal plane, and then the subperiosteal plane beginning 1 cm above the orbital rim. The flap was then transposed into the defect and sutured in place with interrupted sutures (Fig 2). The forehead defect was closed primarily; however, due to tension on the skin edges, a small portion was left to heal by secondary intention.

**Figure 3:**
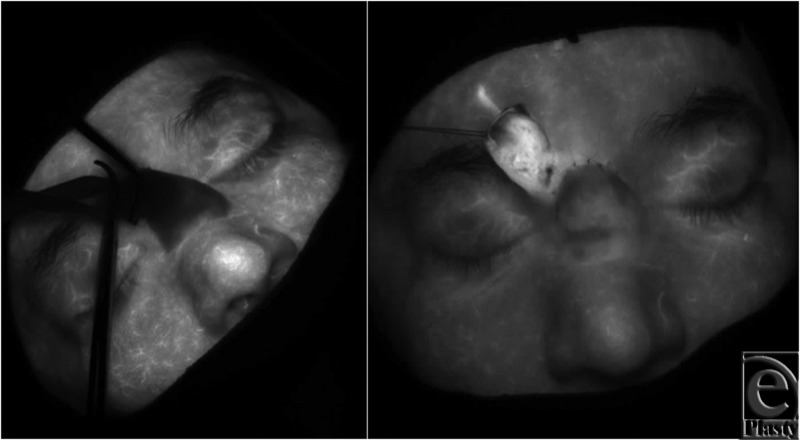
Intraoperative evaluation of forehead flap perfusion after clamping of pedicle and injection of indocyanine green using SPY technology. Fluorescence is demonstrated in transferred flap in spite of pedicle clamping, indicating perfusion to flap. SPY technology subsequently demonstrates continued flap perfusion after division of pedicle and inset of flap.

**Figure 4:**
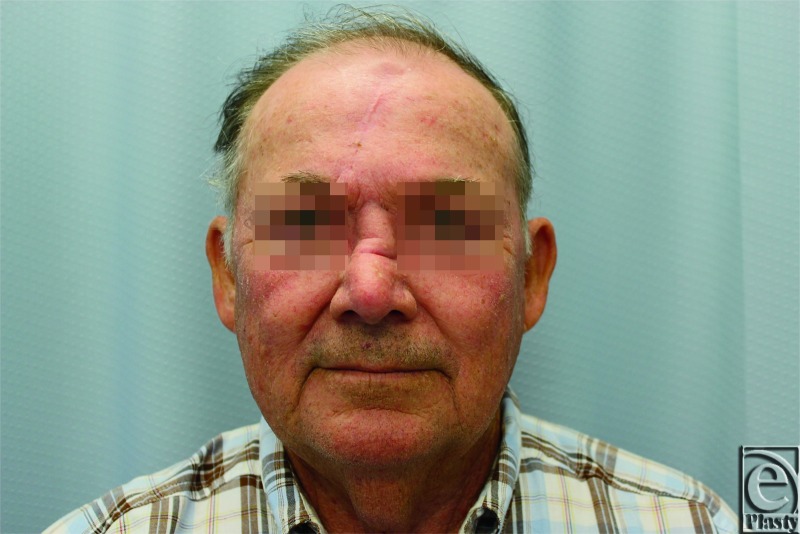
End result of reconstruction.

The patient's postoperative course was uneventful, and 28 days following the first stage, he returned to the operating room for pedicle division and flap inset. Prior to division of the pedicle, perfusion of the flap was assessed using the LifeCell SpyElite System. The pedicle was compressed with both a Penrose drain and a forcep. A 10-mg dose of ICG was administered intravenously and images were captured with the SpyElite System (Fig 3). (There is not currently a consensus as to the dose of ICG that should be administered. The dose chosen was arrived at through discussion with LifeCell representatives as well as extrapolation from previous usage in other reconstructive efforts). Fluorescence was observed throughout the flap and division and inset were performed. A second 10-mg dose of ICG was administered intravenously after inset (23 minutes after the first dose was given) to confirm perfusion and flap viability (Fig 3). Although the tissue appears to have less fluorescence than the surrounding tissues in the first image, the fluorescence will continue to rise as further ICG is perfused into the tissue. The flap has taken up the ICG at a slower rate, likely because the surrounding tissues have a larger network of vascular channels to more quickly perfuse the ICG. This is further confirmed by viewing the second image in Fig 3, which demonstrates an *increase* in fluorescence when compared with the surrounding tissues. This may represent a similar phenomenon, which is that there are less developed vascular channels to drain the flap, leading to a mild degree of venous congestion. The remainder of the patient's operative and postoperative course was uneventful, and he healed without incident (Fig 4)

## DISCUSSION

Large nasal defects remain a reconstructive challenge for the plastic surgeon. Obtaining the appropriate shape, color, texture, and underlying support in large defects is difficult. Often, simple primary closure and small flap advancement are not an adequate option. In these cases, the paramedian forehead flap is a mainstay of reconstruction, which allows the transfer of forehead tissue in an efficient and reliable manner with minimal donor deformity; it creates the most esthetically pleasing reconstruction, both to the recipient nose and the donor forehead.^5^ Traditionally, the forehead flap has been transferred in 2 stages, the first stage for elevation of the flap and the second stage for division of the pedicle and inset of the flap. However, there have been multiple variations and refinements since its inception. These include, but are not limited to, transferring the flap in 3 stages^6^ and incorporating a transverse limb to achieve length and adequate tissue requirements.^7^

Regardless of the technique used, the inset of the flap requires division of the pedicle prior to the completion of reconstruction. After waiting 3 weeks for inosculation of peripheral circulation to the edges of the distal flap, the pedicle is divided. Historically, the assessment of flap perfusion prior to division has been based on clinical judgment and/or the use of fluorescein. Although the rate of partial and full flap necrosis is low, the risk still exists (ranging up to 5.4% in certain series)^8^^-^10 and is exacerbated in patients who are smokers.

Current methods of assessing perfusion are numerous, but previous reviews demonstrate none to be universally accepted.^14^ Conventional methods include monitoring capillary refill, presence or absence of appropriately colored blood when the flap is punctured, and temperature. Also, Doppler Ultrasound has been used as an adjunct to identify arterial perfusion of flaps. These methods are cheaper than others, but their reliability in detecting compromise has come into question. In laser Doppler flowmetry, tissue is illuminated with coherent laser light through a fiber-optic cable. Detection of blood flow and flow velocity up to 8-mm depth is possible. Current laser Doppler systems have integrated temperature sensors to aid in monitoring. Disadvantages are that the system can be sensitive to vibration, motion of the probe, or tissue. The price of the laser Doppler flowmeter ranges upward from $5460. Near-infrared spectroscopy employs the principles of optical spectrometry to measure hemoglobin content and oxygenation in local tissues. Unlike laser Doppler, movements of the probe do not influence the results. The tissue penetration is up to 20 mm. Near-infrared spectroscopy can accurately identify compromise before clinical signs of flap failure, but in the first few postoperative hours, there can also be a slight physiological decrease in oxygen saturation—this can be misunderstood as a vascular complication. The cost for a near-infrared spectroscopy monitoring system is $16,500 for the monitoring box and $150 for the disposable sensor. Microdialysis is a sampling technique that studies the biochemistry of organs or tissues by using a needle and infusion/dialysis system. Tissue can be analyzed on the content of glucose, lactate, pyruvate, and glycerol metabolite concentrations that serve to delineate the biochemical status of the tissue, which is related to perfusion. Microdialysis can detect vascular complications before clinical signs of compromise, but a learning curve is required to optimize the use of the system. Also, it takes 30 minutes to get a reading. The cost for the system is nearly $52,000, and additional costs are up to $570 per flap.^16^

Indocyanine green, a second-generation fluorescent dye, rapidly binds to plasma proteins when injected intravenously. It is taken up from the plasma almost exclusively by the hepatic parenchymal cells and is secreted entirely into the bile. It does not undergo significant enterohepatic recirculation, and it has a half-life of 2.5 to 3.0 minutes. In the absence of capillary protein leakage, it is exclusively distributed within the intravascular space,^11^ which makes it a suitable tracer for vessel perfusion in healthy patients. Indocyanine green absorbs light in the near-infrared range, with a maximum at 805 nm, and fluoresces with a maximum at 835 nm. These absorption and emission values lie in the “optical window” of the skin, where the absorption of intrinsic chromophores, such as hemoglobin and water, is low. Because the skin is relatively transparent to the ICG fluorescence wavelength, the induced fluorescence is not trapped in the skin, and can therefore be recorded by a suitable camera.^12^ The use of a fluorescence marker to assess flap viability is not new, but the availability of a marker with a short half-life, purely vascular distribution, and appropriate excitation wavelength is a significant advance in reconstructive surgery. As demonstrated by Holm et al, cases with arterial spasm, venous congestion, and regional hypoperfusion were revealed by intraoperative ICG videography. Furthermore, there was a strong correlation between intraoperative findings and clinical outcome.^13^

The use of laser-assisted ICG is not without limitations. Indocyanine green should not be given to patients who have an allergy to iodine or shellfish. Currently, the measurement of flap perfusion is not a quantitative measure, but instead, a qualitative one. Perfusion values are given based upon the intensity of the image on the screen, but these values must be interpreted with caution. Variations in hemodynamics, microcirculation, and artifacts are different for each tissue as well as each patient. There are currently ongoing studies that attempt to delineate the numerical values that will correlate with the risk of flap necrosis, but to date, none of these have determined an exact relationship.

## SUMMARY

The ability to evaluate the perfusion and viability of both pedicled and free flaps has been both a source of innovation and frustration for plastic surgeons. We present the use of ICG and the LifeCell SpyElite System to confirm perfusion and viability of a forehead flap prior to division and inset, thereby eliminating the question of flap survival based on clinical judgment alone. This has applicability to a number of different procedures within plastic surgery, including staged procedures such as the Abbe flap for lip reconstruction, the Hughes flap for eyelid reconstruction, the Waltzing tubed pedicle flap, and the groin flap for upper extremity reconstruction. Further studies such as this and others that use laser-assisted ICG (4^,^^15^) must continue to elucidate the possible clinical applications as well as the necessary improvements in technology that will eventually allow us to predict clinical outcome based on the angiographic findings.

## References

[1] Komorowska-Timek E, Gurtner GC (2010). Intraoperative perfusion mapping with laser-assisted indocyanine green imaging can predict and prevent complications in immediate breast reconstruction. Plast Reconstr Surg.

[2] Newman MI, Samson MC (2009). The application of laser-assisted indocyanine green fluorescent dye angiography in microsurgical breast reconstruction. J Reconstr Microsurg.

[3] Sacks JM, Nguyen AT, Broyles JM, Yu P, Valerio IL, Baumann DP (2012). Near-infrared laser-assisted indocyanine green imaging for optimizing the design of the anterolateral thigh flap. Eplasty.

[4] Woodard CR, Most SP (2012). Intraoperative angiography using laser-assisted indocyanine green imaging to map perfusion of forehead flaps. Arch Facial Plast Surg.

[5] Menick FJ (2010). Nasal reconstruction. Plast Reconstr Surg.

[6] Menick FJ (2004). Nasal reconstruction: forehead flap. Plast Reconstr Surg.

[7] Reece EM, Schaverien M, Rohrich RJ (2008). The paramedian forehead flap: a dynamic anatomical vascular study verifying safety and clinical implications. Plast Reconstr Surg.

[8] Little SC, Hughley BB, Park SS (2009). Complications with forehead flaps in nasal reconstruction. Laryngoscope.

[9] Menick FJ (2002). A 10-year experience in nasal reconstruction with the three-stage forehead flap. Plast Reconstr Surg.

[10] Uchinuma E, Matsui K, Shimakura Y, Murashita K, Shioya N (1997). Evaluation of the median forehead flap and the nasolabial flap in nasal reconstruction. Aesthetic Plast Surg.

[11] Ishihara H, Matsui A, Muraoka M, Tanabe T, Tsubo T, Matsuki A (2000). Detection of capillary protein leakage by indocyanine green and glucose dilutions in septic patients. Crit Care Med.

[12] Holm C, Mayr M, Höfter E, Becker A, Pfeiffer UJ, Mühlbauer W (2002). Intraoperative evaluation of skin-flap viability using laser-induced fluorescence of indocyanine green. Br J Plast Surg.

[13] Holm C, Tegeler J, Mayr M, Becker A, Pfeiffer UJ, Mühlbauer W (2002). Monitoring free flaps using laser-induced fluorescence of indocyanine green: a preliminary experience. Microsurgery.

[14] Hirigoyen MB, Urken ML, Weinberg H (1995). Free flap monitoring: a review of current practice. Microsurgery.

[15] Christensen JM, Christensen JM, Baumann DP, Myers JN, Buretta K, Sacks JM (2012). Indocyanine green near-infrared laser angiography predicts timing for the division of a forehead flap. Eplasty.

[16] Smit JM, Zeebregts CJ, Acosta R, Werker PM (2010). Advancements in free flap monitoring in the last decade: a critical review. Plast Reconstr Surg.

